# Fatal orbital cellulitis with intracranial complications: a case report

**DOI:** 10.1186/s12245-018-0211-x

**Published:** 2018-11-22

**Authors:** Sabrina Berdouk, Nirasha Pinto

**Affiliations:** Al Qassimi Hospital, Sharjah, United Arab Emirates

**Keywords:** Orbital cellulitis, Proptosis, Sinusitis, Cavernous sinus thrombosis, Cerebritis, Abscess, Mucormycosis, Ophthalmoplegia

## Abstract

**Background:**

Orbital cellulitis is a relatively uncommon presentation in the emergency department, but orbital cellulitis complicated by intracranial extensions, loss of vision, and death has rarely been reported in the literature.

**Case presentation:**

We report a 40-year-old Pakistani diabetic male complaining of 5 days of bilateral eye pain, proptosis, ophthalmoplegia, headache, and fever. A diagnosis of orbital cellulitis with intracranial extension was made. A computed tomography (CT) scan and a magnetic resonance imaging (MRI) were done on the patient and showed unremarkable orbits, extensive sinusitis, frontal abscess, and multiple septic emboli. The patient was admitted and on day 9 deteriorated and died.

**Conclusion:**

Orbital cellulitis associated with intracranial extension is an extremely rare presentation, and the incidence is unknown. The use of contrast-enhanced imaging studies (CT/MRI) early on in the management of suspected orbital cellulitis is supported by the literature. Cerebritis and brain abscesses resulting from orbital cellulitis need advanced care from multidisciplinary teams. Further studies need to be done to provide recommendations on the use and benefit of surgical intervention.

## Background

Orbital cellulitis is an uncommon complication of rhinosinusitis, but the most common source of orbital cellulitis is rhinosinusitis [1]. Some prospective studies placed the incidence to be 1.6/100 000 in children and 0.1/100 000 in adults [1]. The purpose of our paper is to present the case of a 40-year-old Pakistani male who presented with orbital cellulitis with extensive complications.

## Presentation of the case

A 40-year-old Pakistani male presented to the emergency department (ED) complaining of bilateral dull ocular pain and swelling. The symptoms had started 5 days ago on the left and 2 days ago on the right, and were more severe on the left. The pain was exacerbated by eye movements and associated with lacrimation, redness, and blurry vision. In addition, the patient reported decreased ability to move his eye. He also complained of intermittent frontal headaches, occasional nausea, and a sensation of facial “fullness” for the past 5 days.

He had already sought medical attention from a local clinic 3 days prior to presentation and was prescribed regular oral acetaminophen, diclofenac, and domperidone but failed to improve.

He denied any recent trauma. He denied any recent fevers or constitutional symptoms. He had recently been diagnosed with type 2 diabetes mellitus at the same clinic visit but was not taking any medications. His surgical history was unremarkable. He denied any recent travel history or sick contacts. Prior to this illness, he had reportedly normal 6/6 vision. He worked as a carpenter and claimed to have always worn eye protection while working.

On arrival to the ED, his vitals were as follows: oral temperature 38.4 °C, heart rate 100 bpm, BP 160/100 mmHg, and SpO2 of 99% on room air.

On examination, he was alert and oriented to the three spheres. He looked well but uncomfortable.

**Fig. 1:**
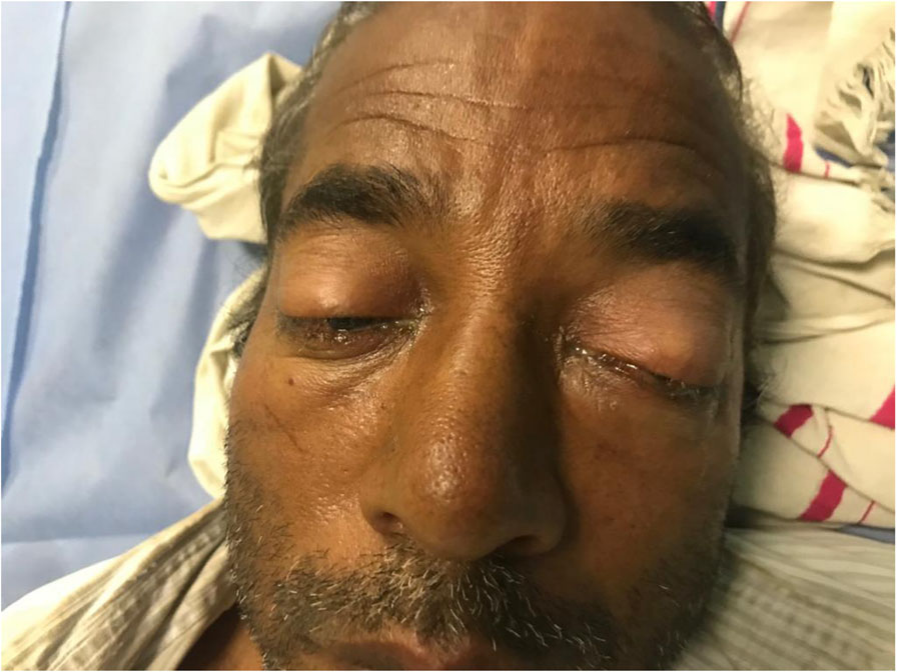
Edema of both eyelids on initial presentation, more extensive on the left

**Fig. 2:**
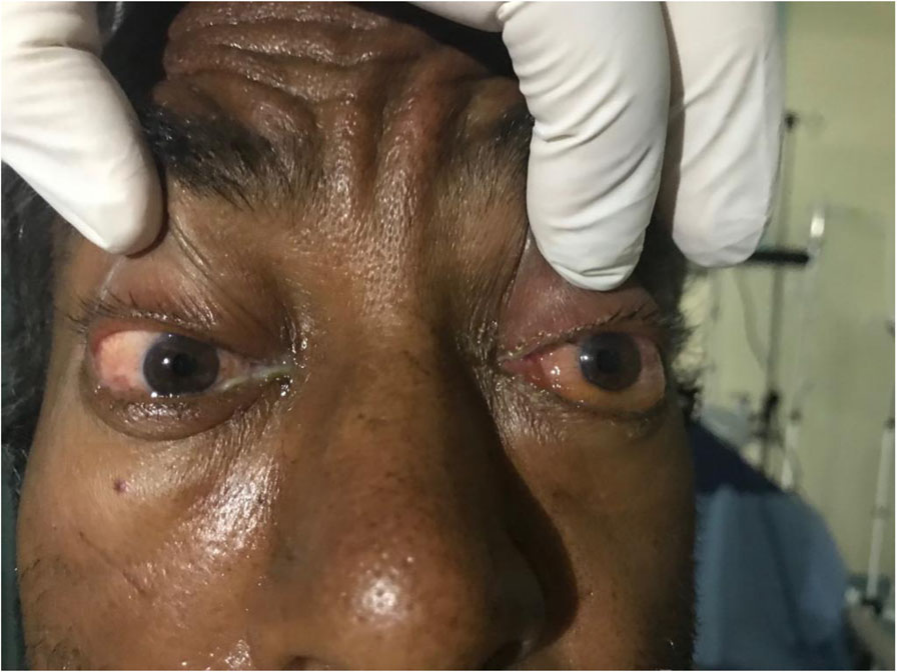
Bilateral chemosis

**Fig. 3:**
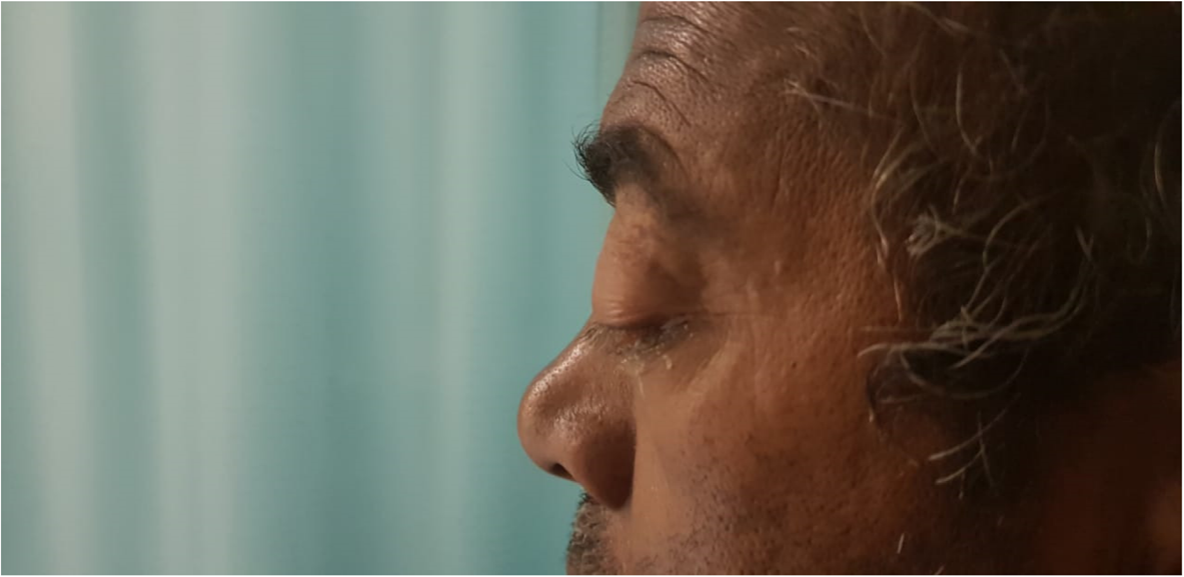
Proptosis of the left eye

**Fig. 4:**
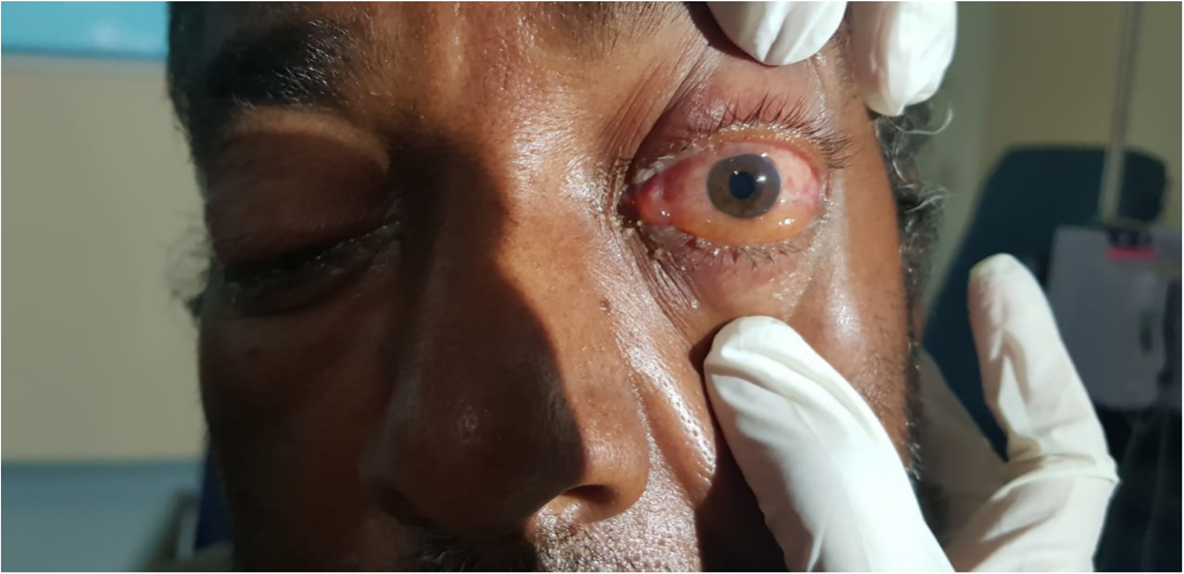
Marked chemosis of left conjunctiva and ciliary injection with matted eye lashes

**Fig. 5:**
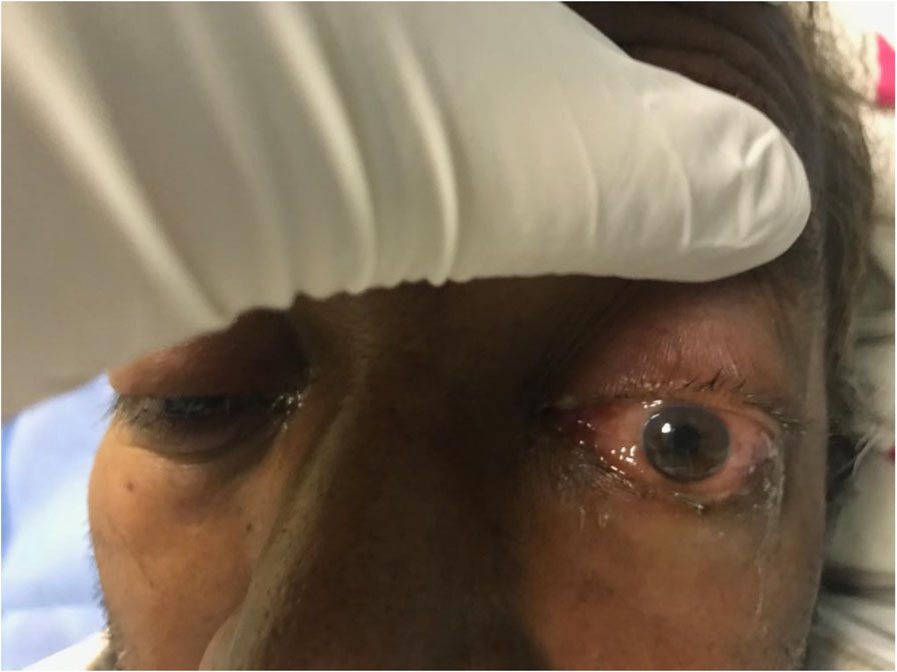
Purulent discharge from the left eye

**Fig. 6:**
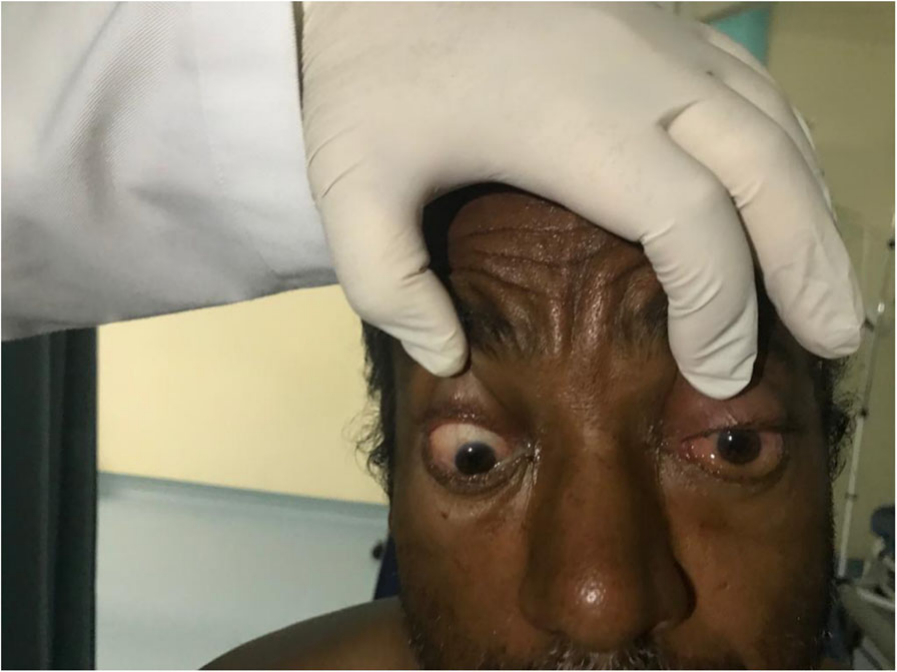
On examining the patient’s eye movements, we noted that he was only able to move his right eye in a downward direction

On inspection, his left eye had significant eyelid edema with purulent discharge, proptosis, chemosis, and ciliary injection, but the cornea was clear (Figs. 1, 2, 3, and 4). He was also exhibiting ophthalmoplegia. On palpation, the left eye was not tense, nor tender. His left pupil was unreactive to light. An afferent pupillary defect was detected on the left eye. He had significant visual loss and was only able to discern flashes of light. His right eye examination also revealed eyelid edema but to a lesser extent than the left, along with purulent discharge, matted eye lashes, erythematous conjunctiva, and a clear cornea (Figs. 4 and 5). His right pupil was reactive to light, but he was able to move his right eye only on a downwards direction (Fig. 6). His vision in the right eye was limited to finger counting at 3 ft.

Both eyes had intraocular pressures of 12 mmHg with normal anterior chambers. The patient was initially seen by the ophthalmologist whose examination reported a cherry red spot on his retina (suggestive of central retinal artery occlusion), along with cloudy swelling on the retina and optic atrophy. The fundoscopic exam of the right eye was normal.

The rest of his physical examination including a neurological exam was unremarkable.

His initial labs revealed a white blood cell count of 11,090 with a 77% neutrophil count. The erythrocyte sedimentation rate (ESR) was 75 mm/h and C-reactive protein (CRP) was 77 mmol/l. The kidney function and serum electrolyte panel were normal.

**Fig. 7:**
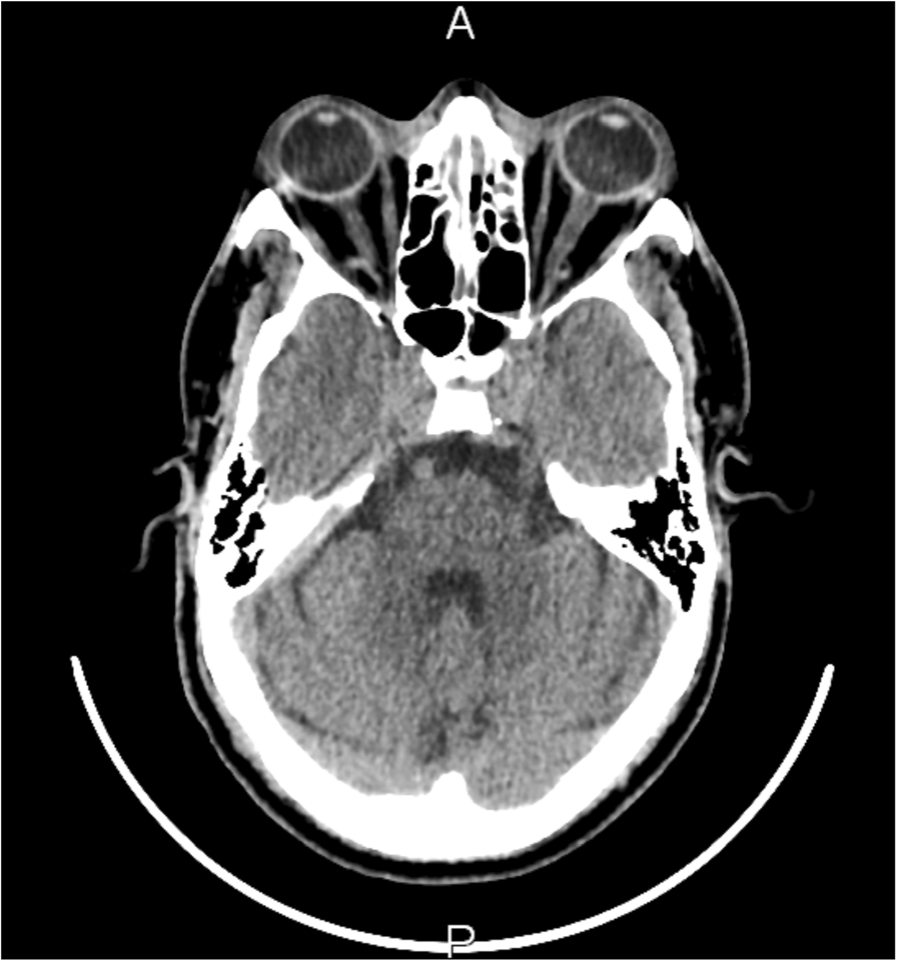
Initial non-contrast CT scan of the orbits, which was reported as unremarkable

**Fig. 8:**
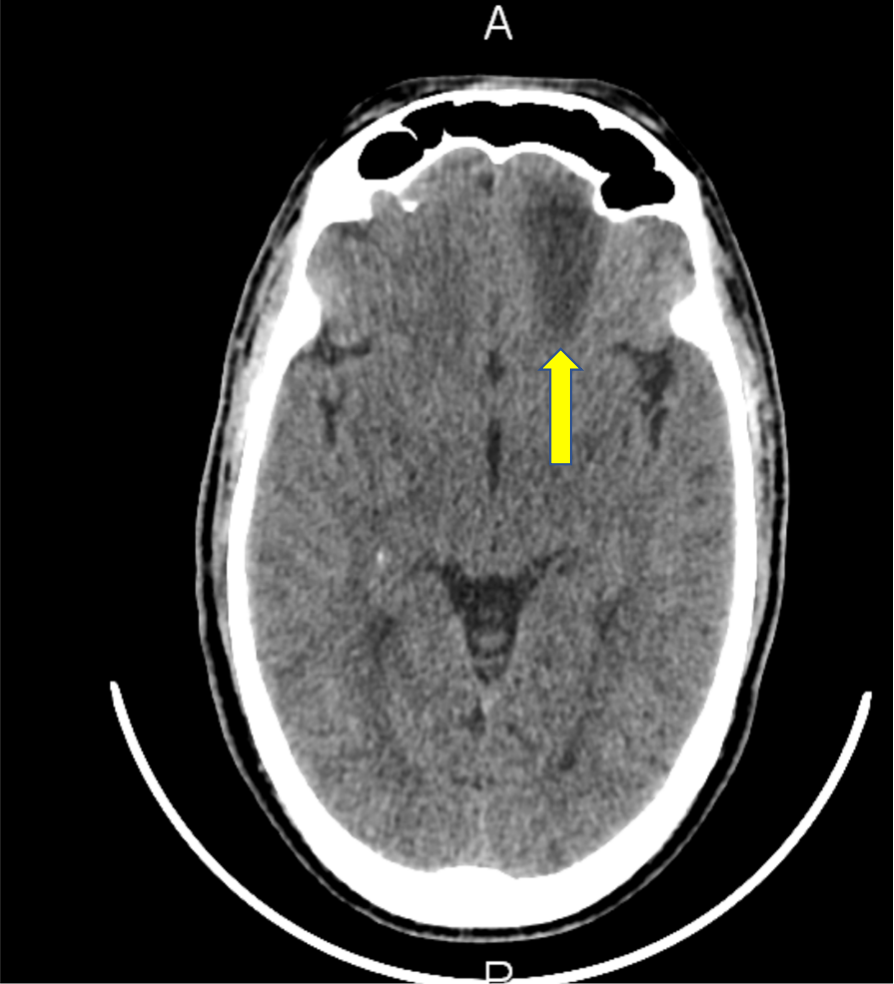
Initial non-contrast CT scan of the brain revealing a hypodensity in the left frontal region of the cerebrum

A clinical diagnosis of orbital cellulitis was made. The patient was started on IV fluids. Early IV antibiotic therapy with IV vancomycin and ceftriaxone was commenced in the emergency department. Initial non-contrast CT scans of the head and orbits were reported as having a left frontal white matter hypodensity posterior to his left eye with unremarkable orbits (Figs. 7 and 8).

**Fig. 9:**
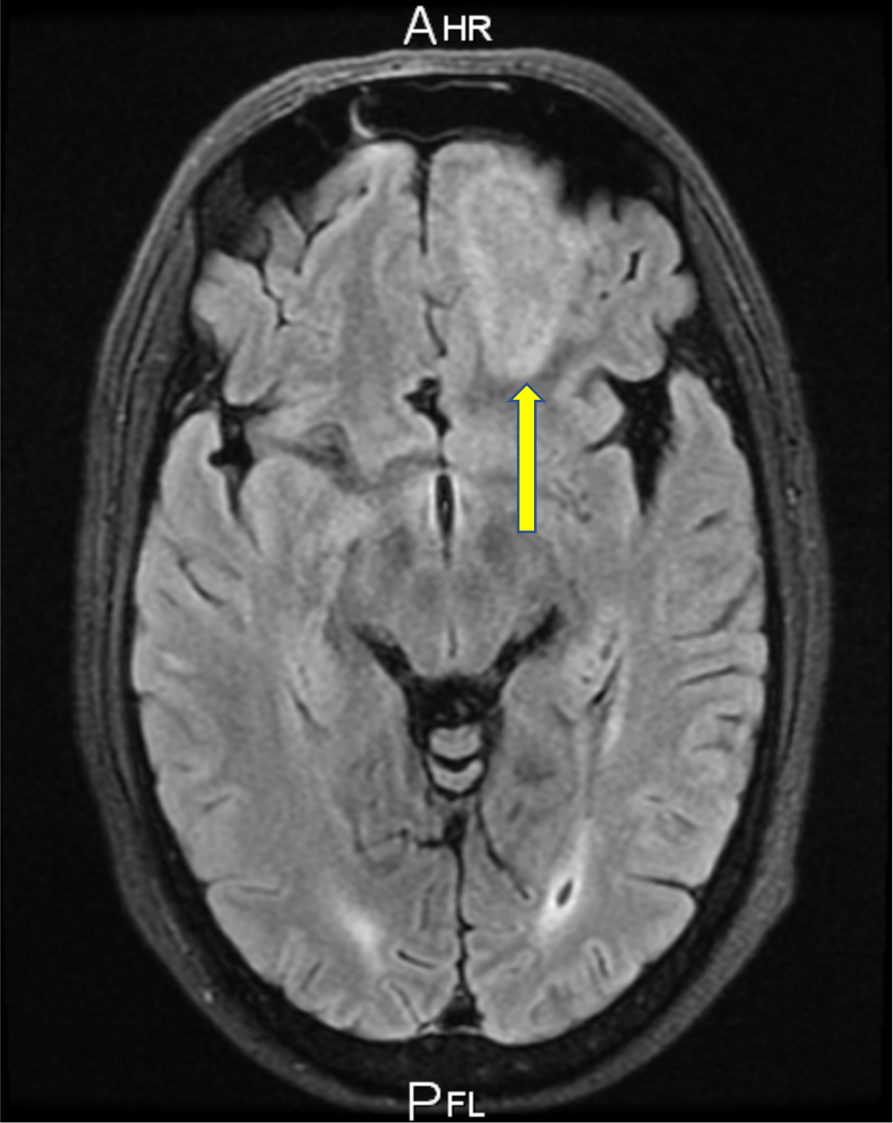
MRI scan of the brain with contrast T2 FLAIR sequence-frontal abscess

**Fig. 10:**
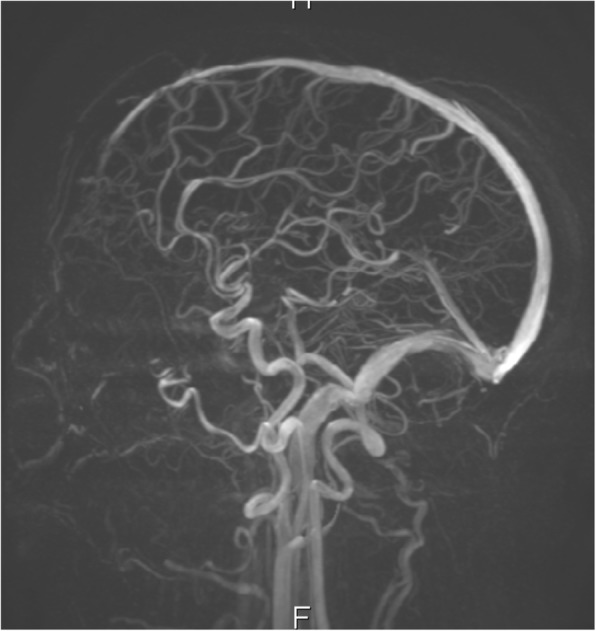
MRV scan of the brain

**Fig. 11:**
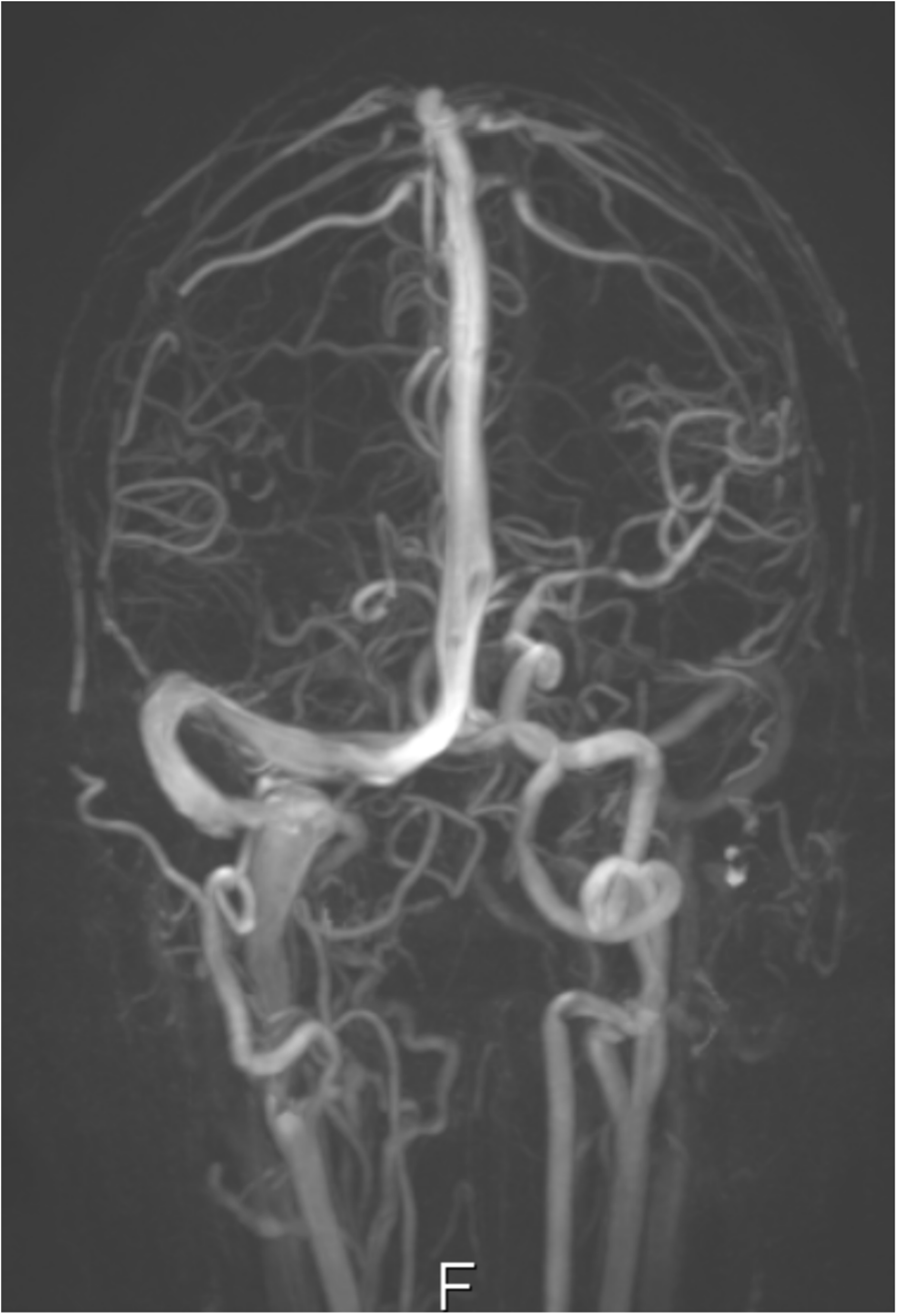
MRV scan of the brain

The patient was admitted under a multidisciplinary team of ophthalmology and neurosurgery. He underwent a further non-contrast CT scan of the sinuses and an MRI of the head with IV contrast which revealed the following: left maxillary, frontal, and sphenoid sinusitis; bilateral ethmoiditis; and a left frontal abscess (Fig. 9). The magnetic resonance venography (MRV) excluded a cavernous sinus thrombus (Figs. 10 and 11).

**Fig. 12:**
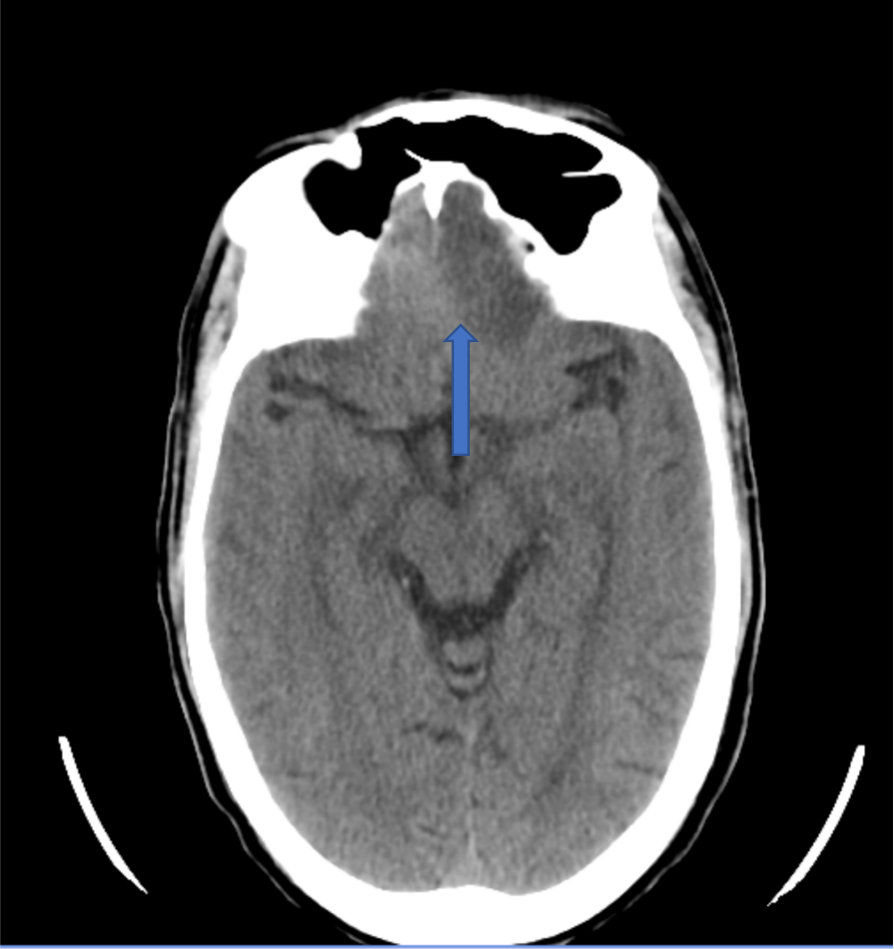
Non-contrast CT scan of the brain revealing a mild midline shift to the right in the frontal region

On day 2, the patient developed progressive left-sided weakness, numbness, and aphasia. He was noted to have become increasingly drowsy. A repeat non-contrast CT scan of the brain revealed a mild midline shift towards the right in the frontal lobe region with no acute ischemic insult (Fig. 12).

**Fig. 13:**
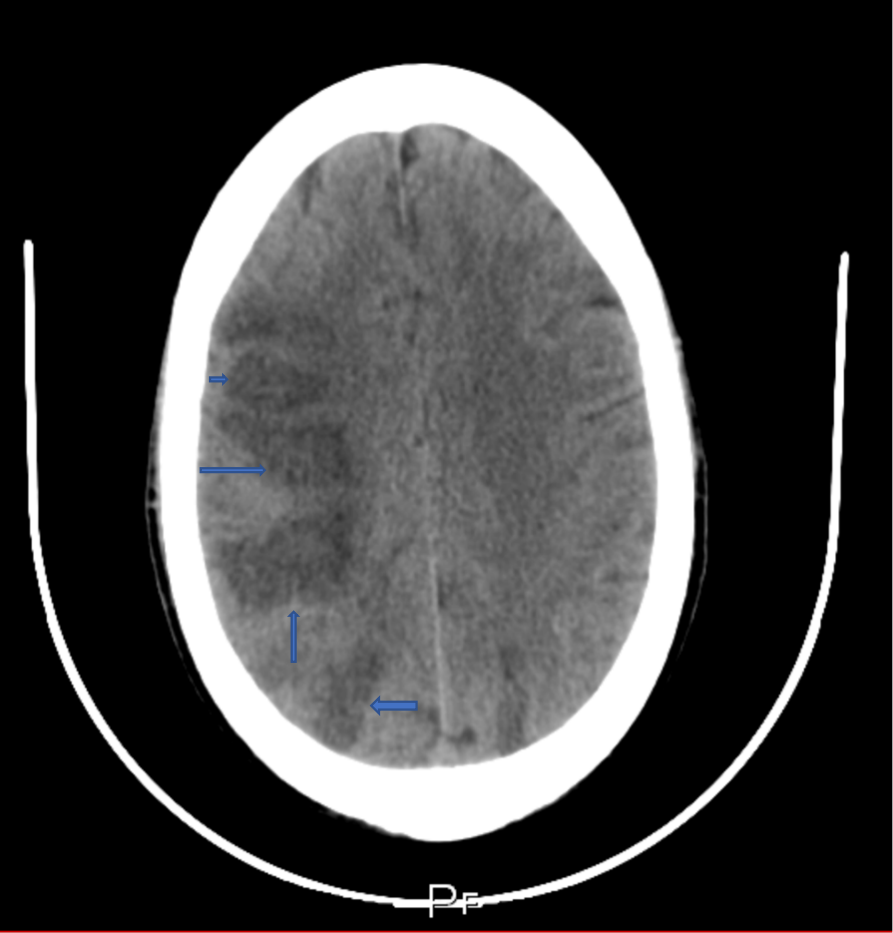
Repeat non-contrast CT scan of the brain with newly developed multiple right parieto-temporal hypodensities

**Fig. 14:**
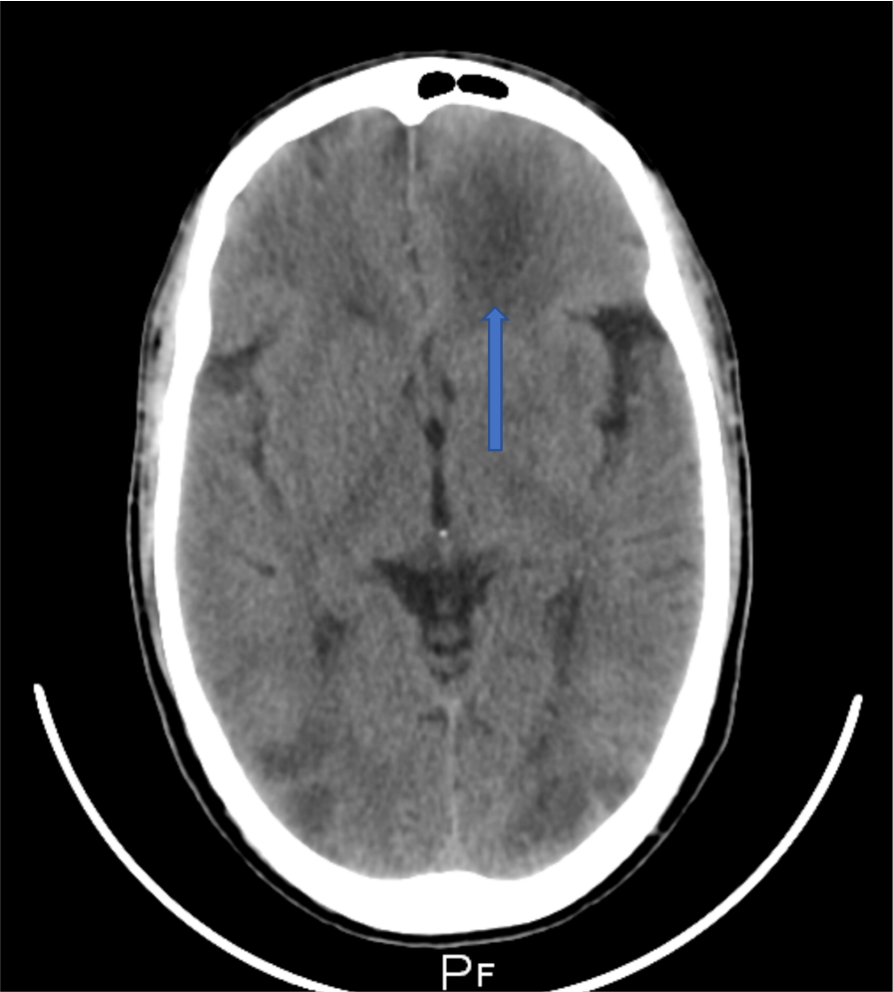
Non-contrast CT scan of the brain revealing further enlargement of the frontal hypodensity

On day 3, the patient’s level of consciousness deteriorated. A third repeat non-contrast CT scan of the brain now revealed multiple newly developed right parieto-temporal and bilateral occipital hypodense lesions, suggestive of septic emboli (Fig. 13). The left frontal hypodense lesion was also noted to have increased in size (Fig. 14).

The patient was placed on mechanical ventilation. He continued to receive full supportive treatment in the ICU while awaiting further investigations.

Blood cultures were negative. Orbital and sinus cultures were not performed. His serology screens (HIV, hepatitis B, and hepatitis C) were negative. A comprehensive vasculitis and thyroid panel screens were negative. The lumbar puncture was also unremarkable. A formal trans-thoracic echocardiogram was negative for thrombi or valvular lesions.

On the ninth day of admission, following a period of progressive decline, the patient arrested and could not be resuscitated.

### Differential diagnosis

Our main differentials for this patient’s eye complaint were pre-septal cellulitis, orbital cellulitis, endophthalmitis, thyroid-associated ophthalmopathy, and severe conjunctivitis. We also suspected trauma, a foreign body imbedded in the eye, particularly given the patient’s history of working as a carpenter. Though he claimed to wear protective gear, hammering and carpentry work are known risks for eye trauma.

Our differentials for the frontal hypodensity on the plain CT included cerebritis, cerebral abscess, or a possible neoplasm.

### Clinical diagnosis

The diagnosis for this patient was orbital cellulitis with intracerebral abscess.

## Discussion

*Orbital cellulitis (OC)* is defined as an infection of the orbital contents posterior to the orbital septum, namely the ocular muscles and fat [1].

A similar yet more benign condition is *pre-septal cellulitis (PSC)*, which is the infection of orbital contents anterior to the orbital septum [1]. The orbital septum is a thin membrane continuing from the periosteum of the orbit to the tarsal plate. Infection of the eye globe itself is termed endophthalmitis [1].

While both PSC and OC may present similarly with eyelid swelling, key signs that indicate OC include proptosis, pain with eye movement, and ophthalmoplegia. If the infection extends far enough to involve the optic nerve, visual impairment may also be present. Since our patient had all these signs, we suspected advanced OC. The ophthalmologist’s findings further pointed out to the diagnosis of an advanced case of orbital cellulitis with optic nerve involvement.

The *most common source* of OC is local spread from a pre-existing adjacent rhinosinusitis due to the close proximity of the sinuses to the orbit. The ethmoid and maxillary sinuses are most frequently implicated, followed by sphenoid and frontal sinuses which develop later in life at around 6 years of age [2]. The thin bony orbital walls can easily be damaged from an adjacent sinus infection causing focal areas of osteitis allowing for spread of pathogens into the orbit. Naturally present focal osseous defects in the orbital walls (i.e., orbital wall fissures and foramina which allow for passage of vessels and nerves) can also contribute to the spread. Another significant factor is the valveless venous drainage of the face. This allows a direct two-way communication between the veins of the face, sinuses, nasal cavity, orbit, pterygoid plexus, and cavernous sinus. Therefore, most orbital and intracranial complications of sinusitis are due to retrograde thrombophlebitis [2].

Other causes of OC include ophthalmic surgery, eye trauma, peribulbar anesthesia, dacryocystitis, and extension from the teeth, middle ear, or face [1].

Our patient’s CT and MRI scans revealed left maxillary, frontal, and sphenoid sinusitis and bilateral ethmoiditis. It is therefore very likely that the source of his OC was from direct extension of the already chronically infected sinuses.

The *most common causative agent* is *Staphylococcus aureus* and streptococci, although atypical bacteria such as Klebsiella, fungal, and polymicrobial infections have been noted in medical literature and are often seen in patients with impaired host defenses.

However, causative agents of OC are generally difficult to identify. They require cultures from the orbit and sinuses which are performed only if surgical intervention is decided. Blood cultures are rarely positive in adults as was the case with our patient [1]. Fungal cultures of the orbits and sinuses should also be performed to rule out invasive fungal infection (i.e., mucormycosis).

Since there was no surgical intervention, neither orbital nor sinus cultures were done. Therefore, the causative agent of our diabetic patient remains unknown. Could invasive mucormycosis cause the rapid deterioration of our patient?

Once orbital cellulitis is suspected, it is generally advised to perform a contrast-enhanced imaging (CT/MRI) of the orbit to identify potential complications, particularly if the patient presents with advanced symptoms, visual impairment, or signs of CNS involvement. Complications include sub-periosteal abscess, orbital abscess, optic nerve involvement leading to vision loss, central retinal artery occlusion, and intracranial extensions (cavernous sinus thrombosis, cerebral abscess).

Our patient’s complaint of headaches, nausea, and fever clued us into suspecting an intracranial involvement. Cerebritis, which is an early form of brain abscess, was detected on the initial non-contrast CT scan of his head.

The cerebritis, in this context, was likely an extension from either the orbital apex or frontal sinusitis. Infection can easily be spread via the valveless venous drainage to the cavernous sinus and dural venous plexus respectively and then on to the cerebral tissues. Frontal sinusitis may also cause a localized osteomyelitis of the calvarium which allows for contiguous spread of infection into the brain. This is supported by multiple studies quoting a higher prevalence of frontal sinusitis in patients found to have intracranial complications [1, 2].

OC associated with an intracranial extension is an extremely rare presentation, and the incidence is unknown. Also, it is approximated that 3–6% of patients hospitalized for sinusitis may develop intracranial complications. However, the true incidence here is hard to determine due to the scarcity of reported cases and because most patients with sinusitis are treated earlier on during their disease stage, often on an outpatient basis [3].

On reviewing the available medical literature, we were able to find only a handful of case reports documenting this rare presentation of OC complicated by brain abscesses. Constantin describes the case of a 12-year-old who presented with features of orbital cellulitis and headache but without exophthalmia or fever. The CT scan revealed a right frontal lobe abscess and maxillary, sphenoidal, and ethmoidal sinusitis. The patient was operated on and initially underwent surgical clearance of the ethmoidal and maxillary sinus collections and an adenoidectomy. This was followed by a craniotomy with excision of the brain abscess and antibiotics. The patient made a complete recovery [4]. Taşdemir describes a fatal case of a 60-year-old man who presented with headache and features of orbital cellulitis, who later develops agitation and found on MRI to have 3–4 mm subdural collection in the right temporal fossa. The patient failed to respond to therapy and died [5]. Yeh describes a 17-year-old boy who presented with fever, headache, drop attacks, and 1-month history of periorbital pain and swelling. He was initially diagnosed with sinusitis and was treated with functional endoscopic sinus surgery and antibiotics. The patient returned a month later with persistent symptoms and found to have a multilobulated lesion in the right frontal lobe. He underwent a bicoronal craniotomy and made a full recovery [6]. Traficante describes a 36-year-old man presenting with left eyelid swelling, headache, and drowsiness. He was seen for a similar complaint 2 weeks prior and had been diagnosed with a periorbital abscess and discharged on oral antibiotics. On the repeat visit, he was found to have altered mental status not consistent with his baseline, with indifference to his condition and flat affect. CT studies revealed rim-enhancing fluid collections in both frontal lobes, suggestive of bi-frontal abscesses. The patient underwent surgery to drain the brain abscess, as well as the sinuses and eyelid abscess. On follow-up, he was noted to have made a full recovery [7].

Once orbital cellulitis is suspected, the patient requires urgent intravenous antibiotic therapy. A frequently used regime includes vancomycin (for MRSA coverage) plus ceftriaxone. If intracranial extension is suspected, metronidazole should be started to provide coverage against anaerobes [8].

In patients failing to improve or those with worsening symptoms, surgical management may be initiated. This involves performing surgical biopsies to identify the causative agent and excision/draining of any infective foci. As the above case reports show, patients with brain abscesses secondary to OC often undergo multiple surgeries—ENT teams operate the sinuses, ophthalmologists operate upon collections in the eye, and neurosurgeons drain cerebral abscesses.

Our patient was treated with IV antibiotics and insulin therapy along with supportive management. He was placed on DVT prophylaxis and underwent numerous investigations to gauge the extent of his illness.

Another complication that we suspected clinically was cavernous sinus thrombosis. Features that would support this include the fact that our patient’s right eye had begun showing symptoms similar to the left eye but 2 days later. The cranial nerves III, IV, V1, V2, and VI pass through the cavernous sinus and supply both sides of the face. An infection within the orbit can therefore pass on pathogens via the cavernous sinus (retrograde through the valveless venous drainage system) onto the contralateral eye [1].

An MRV scan done on the day of admission was reported as showing no signs of cerebral venous thrombosis. However, this scan was not repeated in the subsequent days. We speculate that perhaps there may still have been an element of cavernous sinus thrombophlebitis, which would explain the involvement of the contralateral eye.

A unique feature to our patient’s sequelae is the development of drowsiness, slurred speech, and unilateral weakness during the course of his admission. Our patient clinically deteriorated and had to be intubated, and subsequent CT scans of the head had revealed multiple intracerebral insults.

We suspect that our patient’s deterioration was the result of *multiple septic emboli* arising from the infected sinuses, the infected orbits, or even cavernous sinus thrombophlebitis. A complication of this magnitude has so far, to the best of our knowledge, never been reported in the medical literature.

At present, there are no randomized controlled studies or treatment guidelines specific to orbital cellulitis complicated by brain abscess. As this condition is very rare, the prognosis remains unknown.

## Conclusion

Our case highlights that physicians need to have a high index of suspicion with regard to complications when dealing with cases of orbital cellulitis. Our threshold to investigate such patients should be low, especially if they are elderly, immunocompromised, or come from an underprivileged background with poor access to healthcare. We suspect that our patient had a history of long-standing diabetes mellitus which was untreated, and this may have contributed to his rapid deterioration. We recommend using contrast-enhanced imaging studies (CT/MRI) early on in the management of suspected OC. Cerebritis and brain abscesses resulting from orbital cellulitis need advanced care from multidisciplinary teams. Further studies need to be done to provide recommendations on the use and benefit of surgical intervention.

## Abbreviations

CRP: C-reactive protein
CT: Computer tomography
DVT: Deep vein thrombosis
ED: Emergency department
ENT: Ears, nose, and throat
ESR: Erythrocyte sedimentation rate
IV: Intravenous
MRI: Magnetic resonance imaging
MRV: Magnetic resonance venography
OC: Orbital cellulitis
PSC: Pre-septal cellulitis
WBC: White blood cells
